# PRE-TEST: SPEECH THERAPY PROTOCOL FOR COGNITIVE ASSESSMENT.

**DOI:** 10.1192/j.eurpsy.2023.2189

**Published:** 2023-07-19

**Authors:** M. V. Stilpen, D. M. AVEJONAS

**Affiliations:** FONOAUDIOLOGIA, UNIVERSIDADE DE SÃO PAULO, SAO PAULO, Brazil

## Abstract

**Introduction:**

Autism Spectrum Disorders (ASD) refersto a condition where behavioral and social communication aspects are altered, at different levels of impairment. Relating the characteristics of ASD to the united of brain functioning, alterations in the state of alertness or brain wlkefulness are observed; in the reception, analysis and storage of information and elaboration, programming and execution os activities. The speech therapist is the professional capable of evaluating, diagnosing, preventing and intervening in cases of language impairment, however, there are still few instruments for acessible cognitive investigatio that can guide the professional in the elaboration of a more effective therapeutic plan. Such a lack leads to the need for a longer period of evaluation ans intervention. The present study aims to develop a Cognitive Protocol for Speech language Pathology investigation aimed at children with ASD.

**Objectives:**

Elaboration of the Speech-Language Pathology Protocol for cognitive Investigation aimed at children with ASD (PROFOCO-ASD). In this process, a pre-test was developed to identify difficulties observed by parents and guardians in in understanding, vocabulary, perception of changes and in the response time, in search of better affectiveness of the instrument.

**Methods:**

PROFOCO-ASD has been developed as a doctoral thesis by São Paulo University (USP) and is based on literature review, authors experience, pre-test in target audience and panel of experts. It is a cognitive investigation protocol aimed at children aged between 2 and 12 years with a diagnosis of asd. In the pre-tes phase, 10 parents answers the PROFOCO-ASD , in addition to a separate questionnaire containing questions regarding: a. Understanding of the questions. b. Understanding of the vocabulary userd. c. Perception of the child’s changes, according to the questions asked. d. response time.

**Results:**

the results demonstrated the need for changes in the preparation of the questionnaires, in the vocabulary used, in the size of the questionnaire and in the need for guidance on cognitive alterations, so that párents and guardians could identify them.

**Conclusions:**

The Cognitive Speech-language Pathologist pROTOCOL (PROFOCO-ASD) is a intrument aimed at the speech´language patholist capable of proving a means of identifying fundamental cognitive alterations of language development. In search of greater effectiveness, a pre test was applied in wich parents and guardians answered the questions. The results of the pre-test led to a modification of the protocol, wich had now passed though the expert panel stage.

**Disclosure of Interest:**

None Declared

